# Supplementary material for: Multiple communication mechanisms between sensor kinases are crucial for virulence in Pseudomonas aeruginosa
Source: Nat Commun. 2018 Jun 7;9:2219. doi: 10.1038/s41467-018-04640-8 (PMC5992135; doi:10.1038/s41467-018-04640-8)
Supplement: Supplementary file 1 — Supplementary Information [file 41467_2018_4640_MOESM1_ESM.pdf]

Multiple communication mechanisms between sensor kinases are crucial for virulence in *Pseudomonas aeruginosa*

Vanessa I. Francis[1], Elaine M. Waters[2], Sutharsan E. Finton-James[1], Andrea Gori[1], Aras Kadioglu[2], Alan R. Brown[1], Steven L. Porter[1*]

[1]Biosciences, Geoffrey Pope Building, College of Life and Environmental Sciences, University of Exeter, Exeter, EX4 4QD, UK.

[2]Department of Clinical Infection, Microbiology and Immunology, Institute of Infection and Global Health, University of Liverpool, Liverpool, L69 7BE, UK.

***** For correspondence: Dr Steven Porter

Biosciences

Geoffrey Pope Building

College of Life and Environmental Sciences

University of Exeter

Exeter

EX4 4QD

UK

Email: s.porter@exeter.ac.uk

Telephone: +44 1392 722172

Supplementary Note 1

The *retS* point mutations do not affect the *in vivo* stability of RetS protein

To verify that the *retS* point mutations used in this study do not affect RetS protein stability in *P. aeruginosa*, we raised rabbit antibodies (Eurogentec) against purified RetSc for use in Western blotting. However, the native expression level of RetS in the wild-type PAO1 strain was beneath the detection limit of these antibodies (Supplementary Fig. 3, lane 2). For this reason, we used the pJN105 expression vector to overexpress wild-type RetS and its mutant derivatives in the Δ*retS* strain, allowing us to compare their expression levels and test whether there were any unwanted effects of the point mutations on protein stability. When overexpressing wild-type RetS, we detected several RetS derived species (Supplementary Fig. 3, lane 4): a band of ~104 kDa corresponding to the predicted Mw of the RetS monomer; two smaller bands (~85 & 90 kDa), presumably corresponding to the products of proteolytic degradation/turnover of the overexpressed RetS; and a larger band (~210 kDa) of an appropriate size for a dimeric RetS species that had resisted denaturation. The intensity of all of these bands were comparable between the strain expressing wild-type RetS (Supplementary Fig. 3, lane 4) and the strains expressing the various different RetS mutant proteins (Supplementary Fig. 3, lanes 5-10), confirming that the mutant RetS proteins are expressed as stably as the wild-type RetS protein.

Supplementary Figures

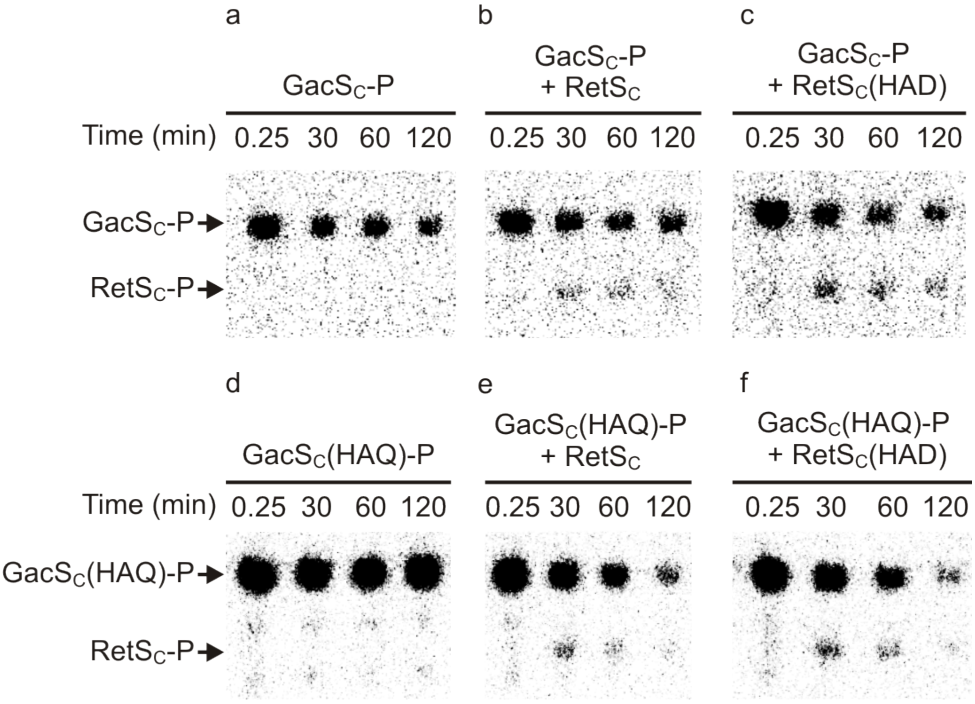

Supplementary Figure 1 | The aspartate residue in the REC1 domain of RetS is not required for phosphotransfer from GacS-P to RetS (mechanism 1). a-c, Phosphorimages of SDS-PAGE gels showing phosphotransfer from GacSc-P to (**a**) buffer control, (**b**) RetSc, and (**c**) RetSc(HAD). **d-f**, Phosphorimages of SDS-PAGE geles showing phosphotransfer from GacSc(HAQ)-P to (**d**) buffer control, (**e**) RetSc, and (**e**) RetSc(HAD). Experiments were repeated 5 times and a representative image shown. The images in panels **d-e** are the same as those used in Fig. 1e-f.

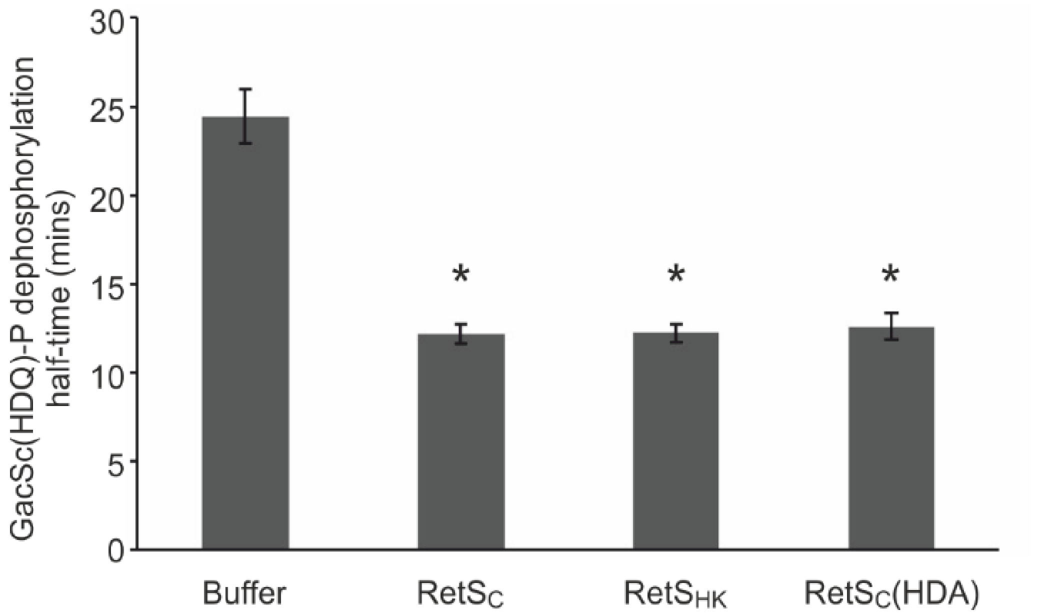

Supplementary Figure 2 | The catalytic core of RetS can stimulate the dephosphorylation of GacSc(HDQ)-P. Dephosphorylation half-time of GacSc(HDQ)-P alone (buffer control) and in the presence of RetS derivatives. Error bars represent SEM (n= 8). * significantly faster than GacSc(HDQ)-P autodephosphorylation (P <0.05, one-way ANOVA).

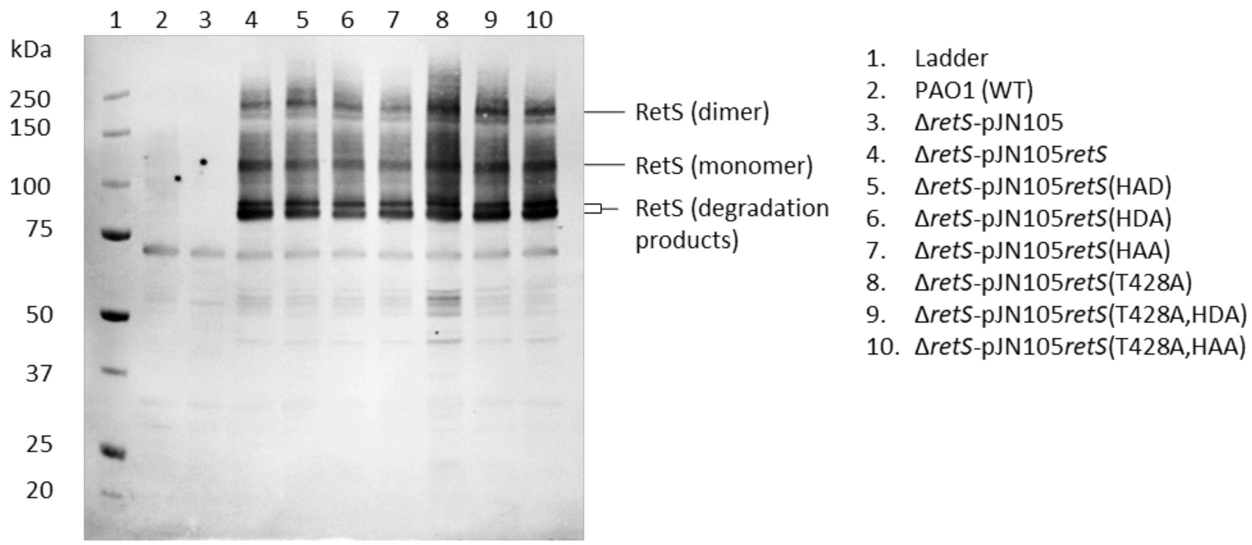

Supplementary Figure 3 | The point mutations within RetS do not affect its expression levels. Western blot showing the overexpression of RetS and various RetS mutant proteins in a *ΔretS* background. Lane 1 - protein ladder. Lane 2 – wild-type PAO1 strain where RetS protein was undetectable owing to its low expression level from the endogenous *retS* promoter. Lane 3 is a negative control with an empty pJN105 vector in the Δ*retS* strain. Lane 4 shows overexpression of wild-type RetS and lanes 5-10 show overexpression of its mutant derivatives from the pJN105 vector in the Δ*retS* strain.

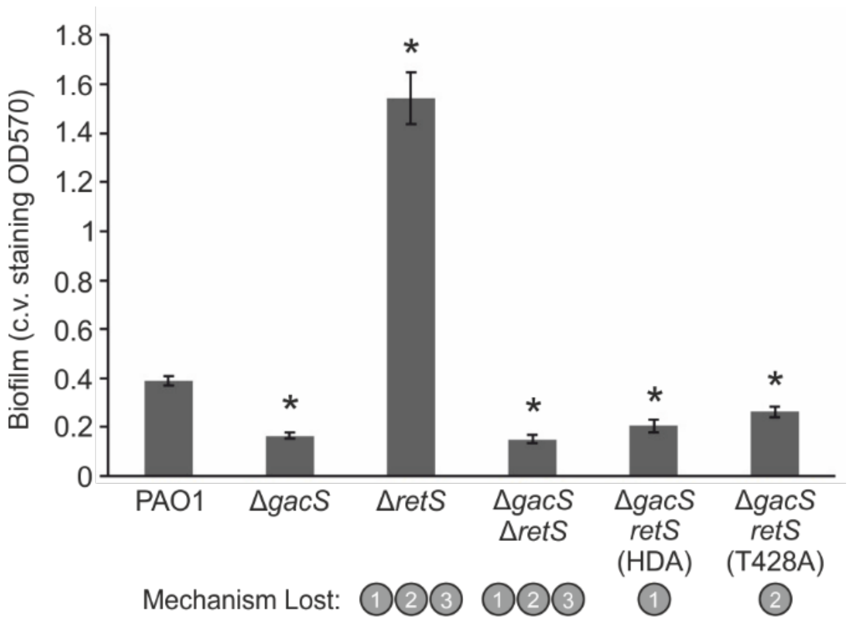

Supplementary Figure 4 | The increased biofilm formation phenotypes of the *retS* point mutants are suppressed by deletion of *gacS*. The graph shows quantification of biofilm formation on peg-lidded 96-well plate by crystal violet staining. Plates inoculated with the mutant strains were incubated for 10 hrs with shaking at 37 °C. Error bars show SEM (three biological repeats each containing 5 technical repeats). * significantly different from PAO1 (P <0.05, one-way ANOVA).

GELID: 20AUG2013_1&2

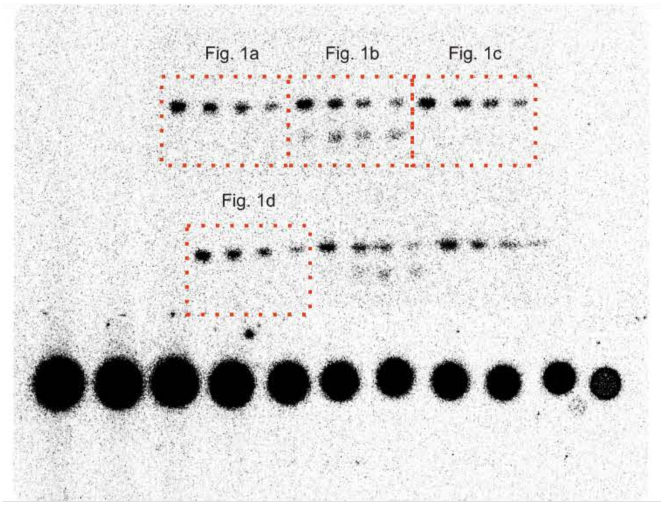

GELID: 05APR12gel3&4

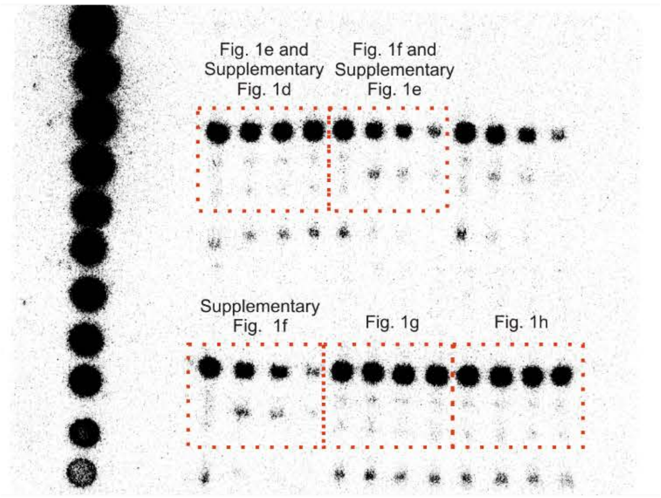

GELID: 05APR12gel1&2

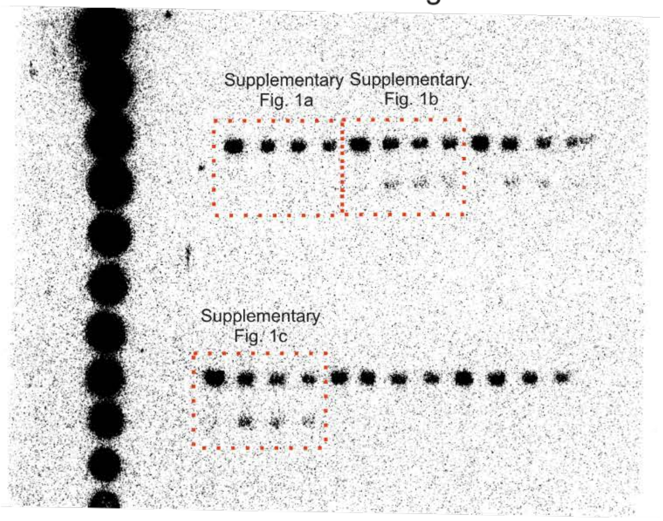

Supplementary Figure 5 | Untrimmed phosphorimages of SDS-PAGE gels comprising Fig. 1a-h and Supplementary Fig 1.

GELID: 020211_3+4

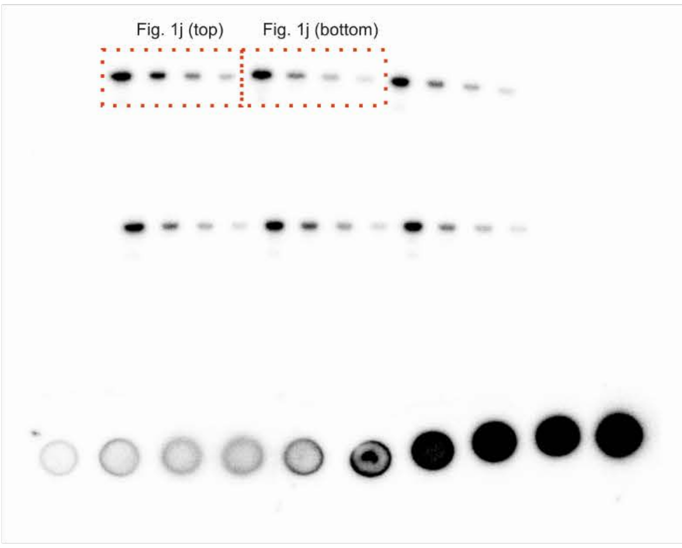

GELID: 16AUG2013_3+4

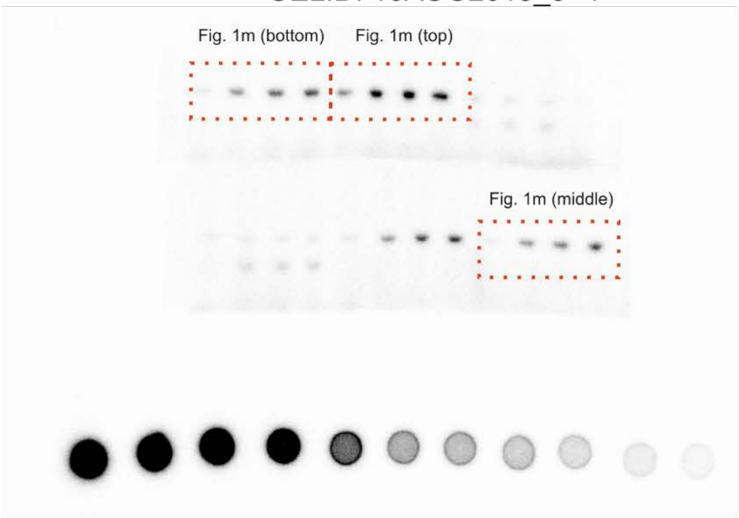

GELID: 110211_1&2

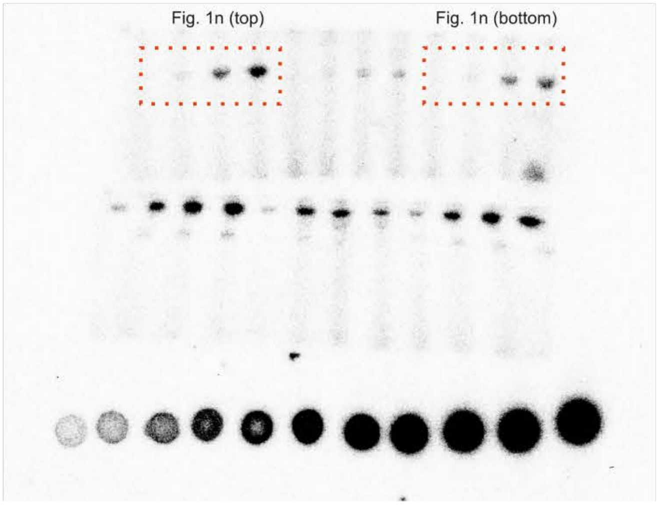

Supplementary Figure 6 | Untrimmed phosphorimages of SDS-PAGE gels comprising Fig. 1j-n.

Supplementary Tables

Supplementary Table 1 | Plasmids and Strains

Plasmid or Strain	Genotype/Description	Source
***E. coli* strains**		
GT115	General cloning strain and expression host	InvivoGen
M15pREP4	Expression host containing pREP4; Km^R	Qiagen
XL1-BLUE	General cloning strain and expression host. *$lacI^q$*; tetracyclin resistance.	Stratagene
**Plasmid vectors**		
pQE60	IPTG-inducible expression vector for *E. coli*. Introduces a C-terminal 6xHis tag; Amp^R	Qiagen
pQE80	IPTG-inducible expression vector for *E. coli*. Introduces a N-terminal 6xHis tag; Amp^R	Qiagen
pREP4	Plasmid containing the *$lacI^q$* gene; Km^R	Qiagen
pEX19Gm	Suicide vector mobilized by *E. coli* S17-1λpir. Confers Gm^R and sucrose sensitivity	[1]
pRK2013	Helper plasmid for mobilisation of non-self-transmissible plasmids. Km^R	[2]
pJN105	Plasmid with an arabinose inducible P_{BAD} promoter for protein expression. Gm^R	[3]
**Plasmids for protein expression**		
pQE60_GacSc	Plasmid for expressing GacSc (the cytoplasmic region of GacS - starting at amino acid 219) in *E. coli*	This study
pQE60_GacSc(HAQ)	Plasmid for expressing GacSc(D715,H859Q) in *E. coli*	This study
pQE60_GacSc(QDH)	Plasmid for expressing GacSc(H293Q) in *E. coli*	This study
pQE60_RetSc	Plasmid for expressing RetSc (the cytoplasmic portion of RetS - starting at amino acid 387) in *E. coli*	This study
pQE60_$RetS_{HK}$	Plasmid for expressing the kinase core (HisKA and HATPase domains; amino acids 387-652) of RetS in *E. coli*	This study
pQE60_RetSc(HAD)	Plasmid for expressing RetSc(D713A) in *E. coli*	This study
pQE60_RetSc(HDA)	Plasmid for expressing RetSc(D858A) in *E. coli*	This study
pQE60_RetSc(HAA)	Plasmid for expressing RetSc(D713A,D858A) in *E. coli*	This study
pQE60_RetS387c (QAA)	Plasmid for expressing RetS387c(H424Q,D713A,D858A) in *E. coli*	This study
pQE60_RetSc (T428A)	Plasmid for expressing RetSc(T428A) in *E. coli*	This study
pQE60_RetSc (T428A_HDA)	Plasmid for expressing RetSc(T428A,D858A) in *E. coli*	This study

**Deletion construct plasmids**		
pEX19GmΔ*retS*	Construct for in-frame deletion of *retS*	This study
pEX19GmΔ*gacS*	Construct for in-frame deletion of *gacS*	This study
pEX19Gm-*retS*(HAD)	Construct for replacing the wild-type *retS* gene in the chromosome with *retS*(D713A)	This study
pEX19Gm-*retS*(HDA)	Construct for replacing the wild-type *retS* gene in the chromosome with *retS*(D858A)	This study
pEX19Gm-*retS*(HAA)	Construct for replacing the wild-type *retS* gene in the chromosome with *retS*(D713A,D858A)	This study
pEX19Gm-*retS*(T428A)	Construct for replacing the wild-type *retS* gene in the chromosome with *retS*(T428A)	This study
pEX19Gm-*retS*(T428A,HDA)	Construct for replacing the wild-type *retS* gene in the chromosome with *retS*(T428A,D858A)	This study
**Complement plasmids**		
pJN*retS*	Full length *retS* in the expression vector pJN105	This study
pJN*retS*(T428A)	Full length *retS*(T428A) in the expression vector pJN105	This study
pJN*retS*(HDA)	Full length *retS*(D858A) in the expression vector pJN105	This study
pJN*retS*(T428A,HDA)	Full length *retS*(T428A,D858A) in the expression vector pJN105	This study
***Pseudomonas aeruginosa* strains**		
PAO1	*P. aeruginosa* wild-type	[4]
Δ*retS*	PAO1 derivative with an in-frame deletion of *retS*	This study
Δ*gacS*	PAO1 derivative with an in-frame deletion of *gacS*	This study
retS(HDA)	PAO1 derivative with *retS*(D858A) in place of wild-type *retS* in the chromosome	This study
retS(HAA)	PAO1 derivative with *retS*(D713A,D858A) in place of wild-type *retS* in the chromosome	This study
retS(T428A)	PAO1 derivative with *retS*(T428A) in place of wild-type *retS* in the chromosome	This study
retS(T428A,HDA)	PAO1 derivative with *retS*(T428A,D858A) in place of wild-type *retS* in the chromosome	This study
ΔretSΔgacS	PAO1 derivative with in-frame deletions of *gacS* and *retS*	This study
retS(HDA)Δ*gacS*	PAO1 derivative with an in-frame deletion of *gacS* and *retS*(D858A) in place of wild-type *retS* in the chromosome	This study
retS(T428A)Δ*gacS*	PAO1 derivative with an in-frame deletion of *gacS* and *retS*(T428A) in place of wild-type *retS* in the chromosome.	This study

Supplementary Table 2 | Primers used in this study

Primer Name	Primer	Plasmid made/other use
**Primers for making the constructs for overexpressing his-tagged proteins**		
GacScF	ATACACGGTCTCACATGGGCAGCAACGAGCTG	pQE60_GacSc
GacScR	CGCGGATCCGAGTTCGCTGGAGTCGAG	pQE60_GacSc
RetSc387F	ATACACGGTCTCACATGCTCACCGAACGCCAGCGG	pQE60_RetSc and pQE60_RetSHK
RetScR	AATCAGATCTGGAGGGCAGGGCGTCGCC	pQE60_RetSc
RetSHKR	AATCAGATCTGTCGAGGTCGGCGGTGGG	pQE60_RetSHK
**Overlap extension primers for introducing point mutations into *gacS* and *retS***		
RetS_TA_ UR	CCGTTCATGGGCGCGCGGATCTCGTGG	pEX19Gm_*retS*(T428A) & pQE60_RetSc(T428A)
RetS_TA_ DF	CCACGAGATCCGCGCGCCCATGAACGG	pEX19Gm_*retS*(T428A) & pQE60_RetSc(T428A)
RetSD713A DF	GACGTGGTCCTGCTCGCCCAGGACATGCCCGGC	pEX19Gm_*retS*(HAD) & pQE60_RetSc(HAD)
RetSD713A UR	GCCGGGCATGTCCTGGGCGAGCAGGACCACGTC	pEX19Gm_*retS*(HAD) & pQE60_RetSc(HAD)
RetSD858A DF	GACCTGGTGCTGATGGCCTGCGAGATGCCGGTTC	pEX19Gm_*retS*(HDA) & pQE60_RetSc(HDA)
RetSD858A UR	GAACCGGCATCTCGCAGGCCATCAGCACCAGGTC	pEX19Gm_*retS*(HDA) & pQE60_RetSc(HDA)
RetSH424Q DF	GGCCAAGATCAGCCAGGAGATCCGCACGCCC	pQE60_RetSc(QDD)
RetSH424Q UR	GGGCGTGCGGATCTCCTGGCTGATCTTGGCC	pQE60_RetSc(QDD)
GacSD715A DF	GACCTGGTCTTCATGGCCGTGCAGATGCCCGGC	pQE60_GacSc(HAQ)
GacSD715A UR	GCCGGGCATCTGCACGGCCATGAAGACCAGGTC	pQE60_GacSc(HAQ)
GacSH859Q DF	GCTCGAGAGGGTCCAGCGGCTGCATGGCGCC	pQE60_GacSc(HDQ)
GacSH859Q UR	GGCGCCATGCAGCCGCTGGACCCTCTCGAGC	pQE60_GacSc(HDQ)
RetSendF	CATGGGTACCCTGGGCATGACCGAACTG	pEX19Gm_*retS*(HDA) & pEX19Gm_*retS*(HAD)
RetSDELDR	CACCAAGCTTGCCAGTGCGCAGACGAACAG	pEX19Gm_*retS*(HDA) & pEX19Gm_*retS*(HAD)
RetS_T428_F	CATGGGTACCCGGTGAGCCTGCTGTGGTTC	pEX19Gm_*retS*(T428A)
RetS_T428_R	CACCAAGCTTGCTTGCTGGCGGAAAGGAAGTC	pEX19Gm_*retS*(T428A)
**Primers for generating Δ*retS* and Δ*gacS* constructs**		
RetSDELUF	CATGGGTACCCGCGCCCATCGTCATCAAGG	pEX19Gm_Δ*retS*
RetSDELUR	CGCGGATCCCACGGCGAAGTCCCTTCGAAGG	pEX19Gm_Δ*retS*
RetSDELDF	CGCGGATCCTCCTGAGGGCAGCGACGTG	pEX19Gm_Δ*retS*
RetSDELDR	CACCAAGCTTGCCAGTGCGCAGACGAACAG	pEX19Gm_Δ*retS*
GacSDELUF	CCGGAATTCCCTGGCTGAACCCGGCGATG	pEX19Gm_Δ*gacS*
GacSDELUR	CGCGGATCCCACACGTCTCTCCGTCGAGCC	pEX19Gm_Δ*gacS*
GacSDELDF	CGCGGATCCTCCACCAGCCTCGACTCCAGCG	pEX19Gm_Δ*gacS*
GacSDELDF	CCCCAAGCTTCTGTCCGGTGCCGATCACTC	pEX19Gm_Δ*gacS*
**Primers for making *retS* complementation plasmid**		
RetS_compF	CCGGAATTCCACGGCCACTTGGCTATAATC	pJN*retS*
RetS_compR	ATCGGAGCTCGAATAGCCGCGTGCGGTTATC	pJN*retS*

**Primers for amplifying genomic DNA regions for sequencing verification**		
GacS_EXT_F	AGGCTGACGATGTCGGACTC	Checking Δ*gacS* mutation
GacS_EXT_R	GCAGGACATGCTGGTAAAGG	Checking Δ*gacS* mutation
RetS_EXT_F	AGAACGTCCAGTTGACCATC	Checking Δ*retS* mutation
RetS_EXT_R	AAAGGCTGTGCCTCTTCTTG	Checking Δ*retS* mutation
RetST428A_EXTF	TCCTGCTGCTGATGTACAAC	Checking *retS*(T428A) mutation
RetST428A_EXTR	AGGACCACGTCGAAGTACTC	Checking *retS*(T428A) mutation
RetS_TA_C	CCACGAGATCCGCGCGCCCATGAACGG	Checking *retS*(HAD) and *retS*(HDD) mutations
RetS_EXT_R	AAAGGCTGTGCCTCTTCTTG	Checking *retS*(HAD) and *retS*(HDD) mutations
**Tetra primer PCR primers for preliminary screening of chromosomal point mutants**		
RetSD713A_T1F	CGCGAGTACTTCGACGTGGTCCTGCTTGC	*retS*(HAD) mutants
RetSD713A_T2R	GCCGGTCATGCCGGGCATGTCCTAGT	*retS*(HAD) mutants
RetSD713A_T3F	ACAACGAAACCTGCCGCAAGGTGCTGGT	*retS*(HAD) mutants
RetSD713A_T4R	GATGATCTTGCTCGGCGCGTTGCTGATC	*retS*(HAD) mutants
RetSD858A_T1F	GGCGACCCAGTACGACCTGGATGCTGATAGC	*retS*(HDA) mutants
RetSD858A_T2R	AAGCCGTCCAGAACCGGCATCTCGCGG	*retS*(HDA) mutants
RetSD858A_T3F	GACTTCCGGATCCTCGTCGCCGAGGACA	*retS*(HDA) mutants
RetSD858A_T4R	AGCTCACGCAATTGCGACAGCTCCACCG	*retS*(HDA) mutants
RetST428A_T1F	TCCTGGCCAAGATCAGCCACGAGATCCTGC	*retS*(T428A) mutants
RetST428A_T2R	ATGCCCAGCACGCCGTTCATGGGAGT	*retS*(T428A) mutants
RetST428A_T3F	CTGTTCGGCACCAAGCAACTCGATCCGG	*retS*(T428A) mutants
RetST428A_T4R	CCAGCTCGATCTGCCCGGACTCCAGCTT	*retS*(T428A) mutants
**Primers for qRT-PCR**		
rsmY_F	TCAGGACATTGCGCAGGAAG	Quantifying RsmY
rsmY_R	ATCCGTGCTACGCCACCATC	Quantifying RsmY
rsmZ_F	CGTACAGGGAACACGCAAC	Quantifying RsmZ
rsmZ_R	ATTACCCCGCCCACTCTTC	Quantifying RsmZ
rpoC_F	CTGTTCAAGCCGTTCATTTTC	Quantifying *rpoC* mRNA
rpoC_R	CTTGATGGTGGTGGCCATA	Quantifying *rpoC* mRNA

Referencesl

1. Hoang, T.T., Karkhoff-Schweizer, R.R., Kutchma, A.J. & Schweizer, H.P. A broad-host-range Flp-FRT recombination system for site-specific excision of chromosomally-located DNA sequences: application for isolation of unmarked *Pseudomonas aeruginosa* mutants. *Gene* **212**, 77-86 (1998).
2. Figurski, D.H. & Helinski, D.R. Replication of an origin-containing derivative of plasmid RK2 dependent on a plasmid function provided in trans. *Proc. Natl. Acad. Sci. USA* **76**, 1648-1652 (1979).
3. Newman, J.R. & Fuqua, C. Broad-host-range expression vectors that carry the L-arabinose-inducible *Escherichia coli araBAD* promoter and the *araC* regulator. *Gene* **227**, 197-203 (1999).
4. Stover, C.K. et al. Complete genome sequence of *Pseudomonas aeruginosa* PAO1, an opportunistic pathogen. *Nature* **406**, 959-964 (2000).
